# Prognostic value of NT-proBNP for myocardial recovery in peripartum cardiomyopathy (PPCM)

**DOI:** 10.1007/s00392-021-01808-z

**Published:** 2021-02-08

**Authors:** J. Hoevelmann, E. Muller, F. Azibani, S. Kraus, J. Cirota, O. Briton, M. Ntsekhe, N. A. B. Ntusi, K. Sliwa, C. A. Viljoen

**Affiliations:** 1 Hatter Institute for Cardiovascular Research in Africa and Cape Heart Institute, Faculty of Health Sciences, University of Cape Town, Cape Town, South Africa; 2 Klinik für Innere Medizin III, Kardiologie, Angiologie und Internistische Intensivmedizin, Universitätsklinikum des Saarlandes, Saarland University Hospital, Homburg (Saar), Deutschland; 3 Division of Cardiology, Groote Schuur Hospital, Faculty of Health Sciences, University of Cape Town, Cape Town, South Africa; 4 Cape Universities Body Imaging Centre, Faculty of Health Sciences, University of Cape Town, Cape Town, South Africa

**Keywords:** Peripartum cardiomyopathy, Heart failure, NT-proBNP, Left ventricular recovery, Risk stratification

## Abstract

**Introduction:**

Peripartum cardiomyopathy (PPCM) is an important cause of pregnancy-associated heart failure worldwide*.* Although a significant number of women recover their left ventricular (LV) function within 12 months, some remain with persistently reduced systolic function.

**Methods:**

Knowledge gaps exist on predictors of myocardial recovery in PPCM. N-terminal pro-brain natriuretic peptide (NT-proBNP) is the only clinically established biomarker with diagnostic value in PPCM. We aimed to establish whether NT-proBNP could serve as a predictor of LV recovery in PPCM, as measured by LV end-diastolic volume (LVEDD) and LV ejection fraction (LVEF).

**Results:**

This study of 35 women with PPCM (mean age 30.0 ± 5.9 years) had a median NT-proBNP of 834.7 pg/ml (IQR 571.2–1840.5) at baseline. Within the first year of follow-up, 51.4% of the cohort recovered their LV dimensions (LVEDD < 55 mm) and systolic function (LVEF > 50%). Women without LV recovery presented with higher NT-proBNP at baseline. Multivariable regression analyses demonstrated that NT-proBNP of ≥ 900 pg/ml at the time of diagnosis was predictive of failure to recover LVEDD (OR 0.22, 95% CI 0.05–0.95, *P* = 0.043) or LVEF (OR 0.20 [95% CI 0.04–0.89], *p* = 0.035) at follow-up.

**Conclusions:**

We have demonstrated that NT-proBNP has a prognostic value in predicting LV recovery of patients with PPCM. Patients with NT-proBNP of ≥ 900 pg/ml were less likely to show any improvement in LVEF or LVEDD. Our findings have implications for clinical practice as patients with higher NT-proBNP might require more aggressive therapy and more intensive follow-up. Point-of-care NT-proBNP for diagnosis and risk stratification warrants further investigation.

## Introduction

Peripartum cardiomyopathy (PPCM) is an important cause of pregnancy-associated heart failure and occurs in women towards the end of pregnancy or within the first five months after delivery [1, 2]. Although up to 46% of patients with PPCM recover their left ventricular (LV) function within 6 months, 23% remain with severely impaired LV systolic function and develop chronic heart failure [3].

Despite recent advances in the management of PPCM, predictors of myocardial recovery remain poorly understood. Previous baseline clinical factors that have been shown to influence LV recovery include LVEF, [4–6] LV dimensions, [5, 7, 8] presence of LV thrombus, [7] right ventricular (RV) systolic dysfunction [9] and African-American ethnicity [5, 7, 10, 11]. Identification of predictors of LV recovery could help to risk stratify patients at the time of PPCM diagnosis, and identify those patients that may benefit from more intensive therapy and follow-up.

B-type natriuretic peptide (BNP) and its prohormone, N-terminal B-type natriuretic peptide (NT-proBNP), are released in response to cardiac wall stress, [12, 13] and are important biomarkers in the diagnosis of heart failure [14]. However, the role of natriuretic peptides is predominantly to rule out heart failure. Previous studies have shown that NT-proBNP is elevated at the time of diagnosis of PPCM [15, 16] and a diagnosis of PPCM is unlikely if a patient presents with BNP < 100 pg/ml or NT-proBNP < 300 pg/ml [2]. Furthermore, NT-proBNP is useful in differentiating healthy postpartum women from those with PPCM or pre-eclampsia [6, 15, 17].

While elevated NT-proBNP levels have been shown to predict mortality and cardiovascular events in patients with heart failure, even amongst those who were asymptomatic, [18] little is known about the prognostic value of NT-proBNP amongst patients with PPCM. In this study, we aimed to assess whether NT-proBNP could serve as predictor of LV recovery in PPCM.

## Methods

### Study design and recruitment

Women with PPCM, seen at the dedicated Cardiomyopathy Clinic at Groote Schuur Hospital, were recruited between 2013 and 2018. Patients were referred from primary or secondary care facilities or within the tertiary/quaternary hospital, and assessed by a team of cardiologists and heart failure specialists.

Inclusion criteria included: (1) primary diagnosis of PPCM, i.e. documented clinical evidence of LV systolic dysfunction towards the end of pregnancy or during the first five months postpartum; (2) no other identifiable causes of heart failure; (3) LVEF ≤ 45% on presentation confirmed by transthoracic echocardiography. Exclusion criteria were: (1) patient unable to give informed consent; (2) other identifiable causes of heart failure; and (3) patients younger than 18 years.

This study was approved by the University of Cape Town’s Faculty of Health Sciences Human Research Ethics Committee (HREC ref no R033/2013), and complied with the Declaration of Helsinki. All patients provided written informed consent prior to study entry.

Eligible patients were enrolled at the baseline visit, at which time their medical and obstetric history, New York Heart Association (NYHA) functional class, clinical examination findings and prescribed medication were recorded. All patients had 12-lead electrocardiogram (ECG) and echocardiogram at the baseline visit. Blood was collected at the baseline visit for the measurement of full blood count, renal function and NT-proBNP.

### 12-lead ECG

A resting 12-lead ECG was performed for all patients at baseline using a MAC 5500 HD (GE Healthcare, Chicago, Illinois, USA) machine. The ECG was analysed for heart rate, rhythm, QRS duration, LV hypertrophy (LVH) by Sokolow–Lyon criteria [19], and a QT interval measurement, corrected by Bazett’s formula (QTcB) [20]. Sinus tachycardia was defined as a heart rate of ≥ 100 beats per minute (bpm). A QTcB interval of ≥ 460 ms was regarded as prolonged [21].

### Echocardiographic assessment

Echocardiography was performed at the time of diagnosis and at follow-up. Two-dimensional and targeted M-mode echocardiography with Doppler colour flow mapping were performed using either a Philips CX50 (Philips, Amsterdam, Netherlands) or a VIVIDi (General Electric Company, Fairfield, Connecticut, USA) echocardiography machine. LV dimensions (i.e. LV end-diastolic diameter [LVEDD] and LV end-systolic diameter [LVESD]) and global systolic function (LV ejection fraction [LVEF]) were measured according to the guidelines endorsed by the American Society of Echocardiography [22].

### Blood tests

Blood samples were taken at the baseline visit. Full blood count and renal function were analysed by the National Health Laboratory Service (NHLS) at Groote Schuur Hospital. For NT-proBNP testing, plasma or serum was separated by centrifugation and aliquots were stored at − 80 °C. NT-proBNP was measured for each participant using the BNP Fragment EIA kit (Biomedica; Vienna, Austria). NT-proBNP values were converted to pg/ml as recommended by current clinical practice guidelines.

### Outcome

We considered two separate echocardiographic measures for LV recovery, i.e. LVEDD < 55 mm and LVEF ≥ 50% within the 12-month follow-up period. Patients who did not fulfil these echocardiographic criteria at follow-up echocardiogram, and those who died within the study period, were considered to have no LV recovery.

### Statistical analysis

Data were collected on Research Electronic Data Capture (REDCap Version 9.5.13), a secure electronic database hosted by the University of Cape Town [23], before being exported to Stata (Version 14.2, StataCorp, College Station, TX, USA) for statistical analysis. Descriptive statistics were used to summarise data. Distribution of data was determined by Shapiro–Wilk test. Continuous variables were summarised as means with standard deviations (SD) for parametric data or median with interquartile range (IQR) for non-parametric data. Categorical variables were expressed as frequencies and percentages. Considering the median NT-proBNP at baseline, we used a cut-off value of 900 pg/ml as a dichotomous variable according to which patients were stratified. Where appropriate, we used a Kruskal–Wallis or Wilcoxon rank-sum test (for continuous variables) and chi-squared or Fisher’s exact test (for categorical variables), to compare outcome measures at follow-up, and whether patients had an initial NT-proBNP of ≥ or < 900 pg/ml. Univariable and multivariable logistic regression analyses were done to determine the association between NT-proBNP value of ≥ 900 pg/ml at presentation and LV recovery (i.e. LV dimension and systolic function) at follow-up. NT-proBNP was adjusted for age and BMI. A *p* value of < 0.05 was considered to indicate statistical significance.

## Results

This cohort of 35 women with PPCM had a mean age of 30.0 ± 5.9 years (Table 1). Almost half of the cohort (45.7%) was multiparous (parity > 3). At the time of diagnosis, 40% had a NYHA functional class III or IV. Overall, the patients presented with a mean heart rate of 90.6 ± 19.6 bpm and a median systolic and diastolic blood pressure of 112 mmHg (IQR 105—138) and 76 mmHg (IQR 70—85), respectively. On echocardiography, the median LVEF was 31% (IQR 24—39), with an LVEDD of 58 mm (IQR 53—64) and LA diameter of 35 mm (IQR 33—39). The median NT-proBNP at the time of diagnosis was 834.7 pg/ml (IQR 571.2—1840.5). The median Hb was 11.9 g/dL (IQR 9.9—12.9), and there was no renal impairment. By the time of discharge, heart failure therapy consisted of beta-blocker (94.3%), angiotensin converting enzyme (ACE)-inhibitor or angiotensin receptor blocker (ARB) (80%), mineralocorticoid-receptor antagonist (MRA) (45.7%) and loop diuretics (91.4%). The dopamine agonist, bromocriptine, was prescribed to 41.1% of the patients in this cohort.

**Table 1:** Baseline characteristics (including demographic, clinical, therapeutic, electrocardiographic and echocardiographic characteristics) predicting recovery of LV dimensions and systolic function with one year

		All	LV dimensions			LV systolic function		
			Recovery within 1 year (LVEDD < 55 mm)	Non-recovery within 1 year (LVEDD ≥ 55 mm)	*P* value	Recovery within 1 year (LVEF ≥ 50%)	Non-recovery within 1 year (LVEF < 50%)	*P* value
		N = 35	N = 18	N = 17		N = 18	N = 17	
Age (years)	Mean ± SD	30.0 ± 5.9	29.7 ± 6.9	30.3 ± 4.8	0.278	30.1 ± 6.8	29.8 ± 5.0	0.692
BMI (kg/m^2^)	Mean ± SD	25.3 ± 4.9	24.38 ± 4.5	26.31 ± 5.27	0.192	24.6 ± 4.8	26.1 ± 5.1	0.222
Breastfeeding (months)	Median (IQR)	16 (5–30)	16 (4–30)	24.5 (5–17)	0.972	16 (6–30)	24 (3–36)	0.778
Parity 1	N (%)	7 (20)	3 (16.67)	4 (23.5)	0.463	2 (11.1)	5 (29.4)	0.420
Parity 2	N (%)	12 (34.3)	8 (44.4)	4 (23.5)		7 (38.9)	5 (29.4)	
Parity ≥ 3	N (%)	16 (45.7)	7 (38.9)	9 (52.9)		9 (50)	7 (41.2)	
NYHA FC III or IV	N (%)	14 (40)	6 (33.3)	8 (47)	0.407	4 (22.2)	10 (58.8)	0.041
SBP (mmHg)	Median (IQR)	112 (105–138)	110 (101—138)	120 (108–133)	0.079	115 (101–138)	110 (108–120)	0.656
DBP (mmHg)	Median (IQR)	76 (70–85)	70 (70–85)	80 (70–80)	0.214	75 (70–85)	76 (70––80)	0.947
Heart rate (per min)	Mean ± SD	90.6 ± 19.6	83 ± 15	98 ± 22	0.025	85 ± 15	97 ± 22	0.072
Sinus tachycardia	N (%)	11 (31.4)	2 (11)	9 (52.9)	0.012	2 (11.1)	9 (52.9)	0.012
QRS width (ms)	Median (IQR)	85 (80–92)	84 (79–89)	86 (82—94)	0.383	84 (80–94)	86 (80–92)	0.967
LVH (Sokolow-Lyon)	N (%)	9 (25.7)	5 (28)	4 (23)	1.000	4 (22.2)	5 (29.4)	0.711
QTcB (ms)	Median (IQR)	455 (423–470)	443 (414–456)	470 (453–480)	0.030	448 (416–474)	463 (436–470)	0.572
Left atrial diameter (mm)	Median (IQR)	35 (33—39)	35 (33—37)	36 (34—44)	0.235	35 (33—37)	35 (34—41)	0.408
LVEDD (mm)	Median (IQR)	58 (53—64)	57 (51—62)	60 (55—65)	0.137	59.5 (51—64)	57 (55—65)	0.364
LVEF (%)	Median (IQR)	31 (24—39)	33 (27—40)	28 (24—38)	0.330	31 (24—38)	32 (25—39)	0.655
Hb (g/dL)	Median (IQR)	11.9 (9.9—12.9)	11.2 (9.4—12.5)	12.1 (11.5—13.1)	0.137	11.9 (9.9—12.7)	11.9 (10.8—13.1)	0.609
Creatinine (µmol/L)	Median (IQR)	61 (54—72)	61 (54 -67)	64 (53 -77)	0.310	63 (55—70)	61 (49—76)	0.509
IFN-γ (I.U./ml)	Median (IQR)	13.2 (11.8—13.8)	13.2 (10.8—13.6)	13.3 (13.1—13.9)	0.407	13.2 (10.7—13.7)	13.2 (13.1—13.8)	0.390
hs-CRP (mg/l)	Median (IQR)	2.3 (1.0—14.7)	1.7 (1.2—21.1)	4.8 (1—8.2)	0.872	3.3 (1.5—13.6)	1.46 (0.9—16.6)	0.430
NT-proBNP (pg/ml)	Median (IQR)	834.7 (571.2–1840.5)	622.3 (534.9–1000.7)	1020.1 (713.4–1865.4)	0.041	693.2 (543.9–1000.7)	1066.2 (682.5–1962)	0.065
NT-proBNP ≥ 900 pg/ml	N (%)	16 (45.7)	5 (27.8)	11 (64.7)	0.028	5 (31.3)	13 (68.4)	0.028
Loop diuretic	N (%)	32 (91.4)	15 (83.3)	17 (100)	0.229	15 (83.3)	17 (100)	0.229
MRA	N (%)	16 (45.7)	7 (38.9)	9 (53)	0.404	10 (55.6)	6 (35.3)	0.315
ACE-i / ARB	N (%)	28 (80)	15 (83)	14 (82)	1.000	14 (77.8)	15 (88.2)	0.658
Beta-blocker	N (%)	33 (94.3)	17 (94)	16 (94)	1.000	17 (94.4)	16 (94.1)	1.000
Bromocriptine	N (%)	14/34 (41.1)	10/17 (58.8)	4 (23.5)	0.080	9/17 (52.9)	5 (29.4)	0.163

*ACE-i* Angiotensin-converting enzyme inhibitors, *ARB* angiotensin receptor blockers, *BMI* Body mass index, *DBP* diastolic blood pressure, *hs-CRP* high-sensitivity C-reactive protein, *IFN-γ* interferon gamma, *LVEDD* left ventricular end-diastolic diameter, *LVEF* left ventricular ejection fraction, *LVH* left ventricular hypertrophy, *MRA* mineralocorticoid-receptor antagonists, *NT-proBNP* N-terminal pro-B-type natriuretic peptide, *NYHA FC* New York Heart Association Functional Class, *QTcB* corrected QT interval by Bazett’s formula, *SBP* systolic blood pressure

### Recovery of LV dimensions

**Fig. 1:**
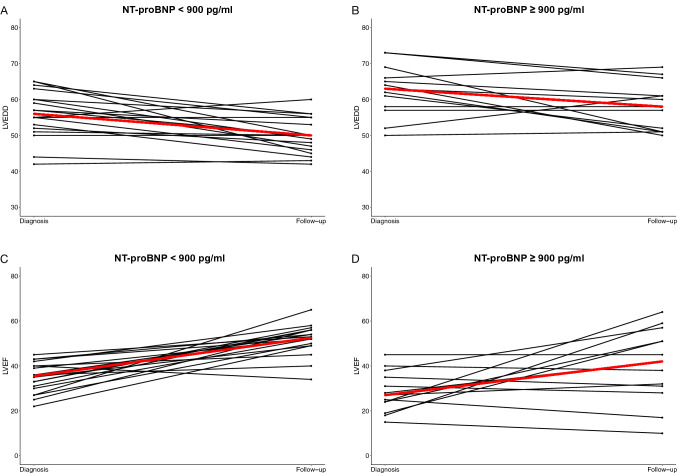
Change of LV dimensions and LV systolic function between diagnosis and one-year follow-up as classified by NT-proBNP < or ≥ 900 pg/ml

Figure 1 shows that most women with PPCM showed overall improvement in LV dimensions at follow-up. However, 18 women (51.4%) recovered their LV dimensions (LVEDD < 55 mm) within the first year after diagnosis. As depicted in Table 1, women who showed recovery of LV dimensions, had a significantly lower heart rate (83 ± 15 vs 98 ± 22 bpm, *p* = 0.025) and were less likely to have sinus tachycardia (11 vs 59%, *p* = 0.012) at initial presentation. The baseline QTc interval was longer, however, in patients who failed to recover their LV dimensions (470 [IQR 453—480] vs. 443 ms [IQR 414—456], *p* = 0.030). The baseline LVEF did not predict recovery of LV dimensions. The initial NT-proBNP was lower in those with recovery of LV dimensions within 12 months (622.3 [IQR 534—1000.7] vs. 1020.1 pg/ml [IQR 713.4—1865.4], *p* = 0.041). As depicted in Fig. 1a and b, the LVEDD decreased at follow visits, regardless of whether the initial NT-proBNP level measured ≥ or < 900 pg/ml (OR 0.42 [95% 0.06—2.95], *p* = 0.380), though the gradient of decline was greater in those with NT-proBNP level measured < 900 pg/ml at baseline. Women with an NT-proBNP of ≥ 900 pg/ml had significantly higher LVEDD at follow-up (58 ± 6.66 vs 49.9 ± 5.05 mm, *p* < 0.001). As shown in Table 2, multivariable regression analysis found that an NT-proBNP of ≥ 900 pg/ml at the time of PPCM diagnosis was predictive of failure to achieve an LVEDD within normal range (LVEDD < 55 mm) at follow-up (OR 0.22 [95% CI 0.05–0.95] *p* = 0.043).

**Table 2:** Univariable and multivariable logistic regression analysis of predictors of recovery of LV dimensions and systolic function within one year

	Univariable regression analysis			Multivariable regression analysis		
	OR	95% CI	*P* value	OR	95% CI	*P* value
Recovery of LV dimensions (LVEDD < 55 mm)						
Age (years)	0.98	0.88—1.10	0.770	1.02	0.89—1.16	0.769
BMI	0.92	0.80—1.06	0.247	0.93	0.79—1.09	0.359
NT-proBNP ≥ 900 pg/ml	0.21	0.05—0.88	0.033	0.22	0.05—0.95	0.043
Recovery of LV systolic function (LVEF ≥ 50%)						
Age (years)	1.01	0.91—1.13	0.865	1.05	0.92—1.19	0.458
BMI	0.93	0.81—1.08	0.345	0.94	0.80—1.10	0.439
NT-proBNP ≥ 900 pg/ml	0.21	0.05—0.88	0.033	0.20	0.04—0.89	0.035

*BMI* body mass index, *LVEDD* left ventricular end-diastolic diameter, *LVEF* left ventricular ejection fraction, *NT-proBNP* N-terminal pro-B-type natriuretic peptide

### Recovery of LV systolic function

Within the first year of follow-up, 51.4% of the cohort recovered their systolic function (LVEF > 50%). Women who did not recover systolic function by 12 months were more likely to present with a NYHA functional class III or IV (58.8 vs. 22.2%; *p* = 0.041) and sinus tachycardia (52.9 vs. 11.1%; *p* = 0.012) at the time of first diagnosis. There was no significant difference in haemoglobin, creatinine or hs-CRP at baseline between women with LV recovery and LV non-recovery.

Although not statistically significant, there was a notable difference in the initial NT-proBNP values between those who recovered their LV function by 12 months and those who did not (693.2 pg/ml [IQR 543.9—1000.7] vs. 1066.2 pg/ml [682.5—1962], *p* = 0.065). More than two-thirds of those who did not recover their LV function within one year presented initially with NT-proBNP levels of ≥ 900 pg/m (68.4 vs. 31.3%, *p* = 0.028). Patients with a baseline NT-proBNP < 900 pg/ml showed more consistent recovery of their systolic function at follow-up (Fig. 1c) (LVEF of 53%, IQR 49–56), whereas there was more variation in LVEF at follow-up amongst those patients with NT-proBNP ≥ 900 pg/ml (LVEF of 42%, IQR 31–51, *p* = 0.045) (Fig. 1d). Indeed, women with NT-proBNP levels of ≥ 900 pg/ml had a lower likelihood of LV functional improvement (OR 0.12 [95% CI 0.02—0.69], p = 0.017). As depicted in Table 2, multivariable regression analysis found NT-proBNP level of ≥ 900 pg/ml to be predictive of persistent impaired systolic function (LVEF < 50%) at follow-up (OR 0.20 [95% CI 0.04—0.89], *p* = 0.035).

### Factors associated with increased NT-proBNP

When adjusted for age and BMI, an NT-proBNP level of ≥ 900 pg/ml remained a significant predictor of recovery of LV dimension (OR 0.22 [95% CI 0.05—0.95], *p* = 0.043) and systolic function (OR 0.20 [95% CI 0.04—0.89], *p* = 0.035) within 12 months (Table 2).

**Fig. 2:**
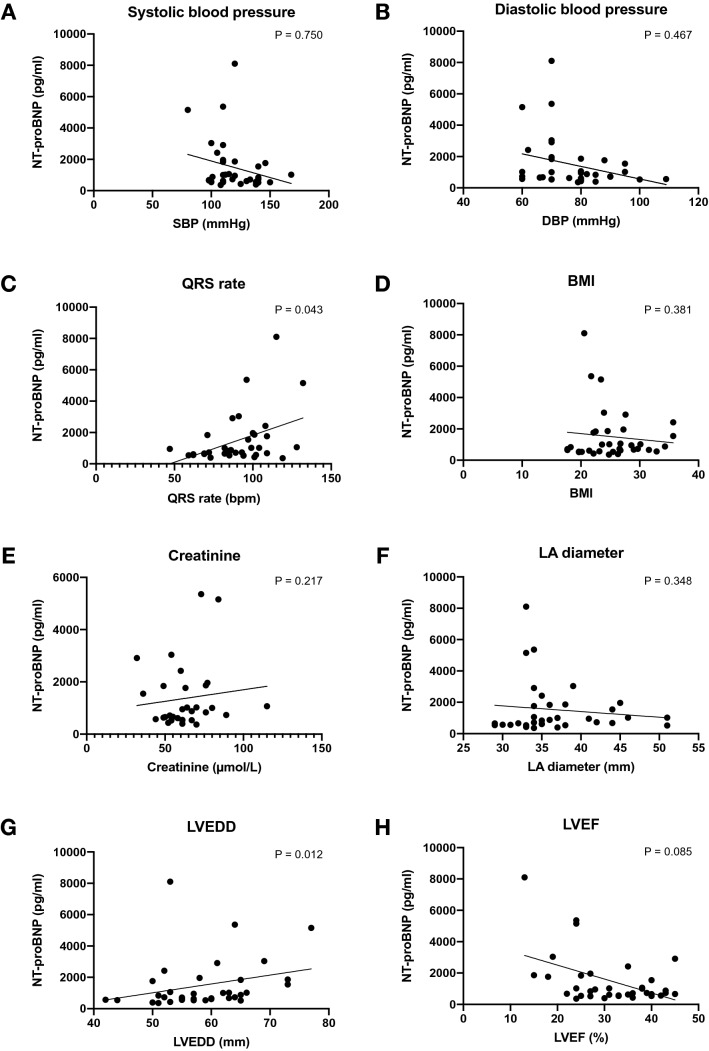
Correlation between NT-proBNP and clinical, biochemical and echocardiographic parameters

There was no correlation between NT-proBNP and blood pressure (SBP *r* =  – 0.06, *p* = 0.751; DBP *r* = -0.127, *p* = 0.467) – Fig. 2. Similarly, NT-proBNP did not correlate with BMI or creatinine. However, there was a modest correlation between NT-proBNP levels and heart rate (*r* = 0.344, *p* = 0.043) and LVEDD (*r* = 0.420; *p* = 0.012).

Table 3 depicts the clinical factors that were associated with higher baseline NT-proBNP levels. Women with NT-proBNP ≥ 900 pg/ml at the time of diagnosis, had a higher heart rate (98 vs 85 bpm; *p* = 0.028), a tendency towards a larger left atrial (LA) diameter (37 vs 35 mm, *p* = 0.091), and significantly increased LVEDD (62.5 vs 55 mm; *p* = 0.027). Furthermore, these patients presented with a significantly lower LVEF (26 vs 35%; *p* = 0.035) at the time of diagnosis. There was no difference in parameters that are known to influence NT-proBNP (i.e. BMI, creatinine).

**Table 3:** Differences in baseline characteristics as classified by NT-proBNP < or ≥ 900 pg/ml

		NT-proBNP < 900 pg/ml	NT-proBNP ≥ 900 pg/ml	*P* value
		N = 19	N = 16	
Age (years)	Mean ± SD	29.1 ± 6.4	31.1 ± 5.2	0.246
BMI (kg/m^2^)	Mean ± SD	24.6 ± 5.2	26.2 ± 4.5	0.289
Breastfeeding (months)	Median (IQR)	20 (6—30)	16 (3—34)	0.832
Parity 1	N (%)	3 (15.8)	4 (25)	0.821
Parity 2	N (%)	7 (36.8)	5 (31.3)	
Parity ≥ 3	N (%)	9 (47.4)	7 (43.8)	
NYHA FC III or IV	N (%)	4 (21.1)	10 (62.5)	0.018
SBP (mmHg)	Median (IQR)	118 (101—138)	111 (107—129)	0.702
DBP (mmHg)	Median (IQR)	76 (70—85)	75 (66—85)	0.738
Heart rate (per minute)	Mean ± SD	85 ± 16.8	98 ± 20.6	0.028
Sinus tachycardia	N (%)	3 (15.8)	8 (50)	0.065
QRS width (ms)	Median (IQR)	84 (80—88)	86 (82—94)	0.451
LVH (Sokolow–Lyon)	N (%)	5 (26.3)	4 (25)	1.000
QTcB (ms)	Median (IQR)	454 (415—470)	459 (436—470)	0.621
Left atrial diameter (mm)	Median (IQR)	35 (32—37)	37 (34—43)	0.091
LVEDD (mm) at diagnosis	Median (IQR)	55 (51—60)	62.5 (55—67.5)	0.027
LVEF (%) at diagnosis	Median (IQR)	35 (27—40)	26 (21.5—36.5)	0.035
LVEDD (mm) at follow-up	Median (IQR)	50 (46—55)	58 (51—61)	0.001
LVEF (%) at follow-up	Median (IQR)	53 (49—56)	42 (31—51)	0.045
Haemoglobin (g/dL)	Median (IQR)	11.7 (9.4—12.9)	12 (10.4—12.7)	0.947
Creatinine (µmol/L)	Median (IQR)	61 (53—67)	64 (54—77)	0.249
IFN-γ (I.U./ml)	Median (IQR)	13.3 (10.7—13.6)	13.2 (13.0—13.8)	0.531
hs-CRP (mg/l)	Median (IQR)	1.7 (1.0—4.9)	4.6 (1.1—39.3)	0.403
Loop diuretic	N (%)	17 (89.5)	15 (93.8)	1.000
MRA	N (%)	7 (36.8)	9 (56.3)	0.251
ACE-i / ARB	N (%)	14 (73.7)	15 (93.8)	0.187
Beta-blocker	N (%)	18 (94.7)	15 (93.8)	1.000

*ACE-i* Angiotensin-converting enzyme inhibitors, *ARB* angiotensin receptor blockers, *BMI* Body mass index, *DBP* diastolic blood pressure, *hs-CRP* high-sensitivity C-reactive protein, *IFN-γ* interferon gamma, *LVEDD* left ventricular end-diastolic diameter, *LVEF* left ventricular ejection fraction, *LVH* left ventricular hypertrophy, *MRA* mineralocorticoid-receptor antagonists, *NT-proBNP* N-terminal pro-B-type natriuretic peptide, *NYHA FC* New York Heart Association Functional Class, *QTcB* corrected QT interval by Bazett’s formula, *SBP* systolic blood pressure

## Discussion

In this study, we show that women with PPCM present with markedly increased NT-pro-BNP levels at the time of diagnosis. In this regard, a baseline NT-proBNP ≥ 900 pg/ml is predictive of failure to recover LV systolic function and dimensions at one-year follow-up. Importantly, NT-proBNP levels are usually not elevated during normal pregnancy or healthy postpartum period [24]. NT-proBNP levels in our cohort were higher than what has been reported for women during normal pregnancy and healthy postpartum period, [15, 24] and those reported for women with pre-eclampsia [17]. This corresponds to what has previously been reported for patients with PPCM in South Africa, Germany and China [6, 15, 16]. The NT-proBNP values seen in this cohort (mean 834.7 pg/ml), however, were lower than those recently reported for the 739 patients included in the European Observational Research Project (EORP) on PPCM, where the median NT-proBNP was 3308 pg/ml [3].

Contemporary heart failure guidelines recommend natriuretic peptides as the biomarker of choice in diagnostic work-up of patients with heart failure [25]. In this regard, the diagnostic role of BNP or NT-proBNP is predominantly to exclude heart failure in peripartum patients. In a study by Malhame et al*.,* a BNP cut-off value of < 111 pg/ml excluded heart failure in pregnant and postpartum women [26]. For PPCM, a threshold of < 100 pg/ml for BNP and < 300 pg/ml for NT-proBNP was proposed to rule out heart failure during pregnancy or the postpartum period [27].

Natriuretic peptides have also been studied for its prognostic value during pregnancy and the postpartum period. Normal BNP levels (< 100 pg/ml) have been shown to have a negative predictive value for adverse maternal events in pregnant women with heart disease [24]. In women with congenital heart disease, BNP levels > 128 pg/ml measured at 20-week gestation predicted adverse cardiovascular events later in pregnancy [28].

The predictive value of NT-proBNP in patients with mild to moderate heart failure was studied in a sub-study of the COPERNICUS trial (n = 1,011) in which patients were stratified according to whether their NT-proBNP levels were above or below the median value of the cohort [29]. Hartmann et al*.* described increased mortality and rehospitalisation for heart failure amongst patients with an NT-proBNP above the median of the cohort [29]. Considering the median baseline NT-proBNP in this cohort, we chose an arbitrary cut-off value of 900 pg/ml according to which our patients were classified. We found that patients with NT-pro-BNP of ≥ 900 pg/ml had significantly higher LVEDD and significantly lower LVEF at one-year follow-up. Although most patients showed some improvement in LVEDD and LVEF at follow-up, women with NT-pro-BNP of ≥ 900 pg/ml were less likely to recover their LV dimensions and systolic function within normal range within one-year follow-up.

When interpreting NT-proBNP levels in heart failure, various clinical factors need to be considered. The level of natriuretic peptides increases with age, and therefore, higher cut-off values are suggested for the elderly. Whereas obesity lowers the concentration of natriuretic peptides, renal disease and atrial arrhythmias (atrial fibrillation (AF) in particular) are associated with higher NT-proBNP levels. None of the patients in this cohort had AF; this was not surprising, as AF has previously been described to be rare in PPCM [30]. Furthermore, the median creatinine was 61 µmol/L in this cohort and there was no significant correlation between creatinine and NT-proBNP. Adjusting for age and BMI, baseline NT-proBNP ≥ 900 pg/ml was a predictor of failure to recover LV dimensions and systolic function within one year in this South African cohort. In a Chinese cohort of 71 patients with PPCM, Li et al*.* reported that NT-proBNP levels of > 1860 pg/ml were associated with a threefold increase in persistent LV systolic dysfunction at follow-up [16]. In contrast, Biteker et al*.* did not find BNP to be predictive of recovery of LV dimensions and systolic function in a cohort of 43 women with PPCM from Turkey [31].

Although our study showed that in most patients, there was an improvement in LV dimensions and systolic function, some remained with an LVEDD > 55 mm and LVEF < 50% at follow-up. We found that women with baseline NT-proBNP ≥ 900 pg/ml were more likely to show no improvement in their LV systolic function at one-year follow-up. This is supported by previous work by Forster et al*.* who reported that NT-proBNP levels were significantly higher in those women who did not show an improvement of at least 10 percentage units (e.g. 25–35%). In their study, NT-proBNP levels remained significantly higher in those who did not improve their LV function [15].

Inflammation, increased levels of oxidative stress and systemic angiogenic imbalance appear to play a crucial role in the pathophysiology of PPCM. Through unknown mechanisms, increased levels of oxidative stress cause a cleavage of prolactin into a 16-kDa fragment, which causes endothelial dysfunction and induces cardiomyocyte apoptosis [32, 33]. Moreover, auto-immune mechanisms have been suggested to be involved in the pathogenesis of PPCM [34, 35]. More severe forms of the disease have been shown to have higher levels of auto-antibodies. In this regard, circulating auto-antibodies against cardiac sarcomeric myosin (MHC) and troponin I (TnI) were also associated with higher levels of NT-proBNP. Indeed, patients with auto-antibodies have been described to have lower rates of LV recovery [35].

Increased NT-proBNP levels are associated with LV remodeling [36]. In this cohort, elevated levels of NT-proBNP tended to be associated with a lower LVEF. However, there was there was a significant correlation between LVEDD and NT-proBNP in this cohort. As expected, those with NYHA functional class III or IV also had higher levels of NT-proBNP, in keeping with a more severe stage of heart failure. Although there was no correlation between systolic or diastolic blood pressure and NT-proBNP, we found a positive correlation between heart rate and NT-proBNP levels. Those patients with sinus tachycardia at time of initial diagnosis, had significantly higher levels of NT-proBNP. This underlines the importance of recognising the presence of sinus tachycardia in patients with heart failure, as sinus tachycardia has previously been reported as a predictor of poor outcome in PPCM [37].

Although not explored in this study, point-of-care (POC) NT-proBNP tests have previously been used in patients with heart failure. The benefit of POC testing is its ease of use, affordability and the immediate availability of the result [38]. Therefore, NT-proBNP bedside testing in patients with PPCM is an exciting prospect, especially in health care centres in low- and middle-income countries, where echocardiography is not readily available [14]. POC NT-proBNP testing may potentially assist the primary care physicians with diagnosis of PPCM, risk stratification, and timely referral [39]. The role of POC NT-proBNP testing, however, still needs to be studied in PPCM.

## Limitations

As this is a single-centre study conducted at a tertiary hospital, where the most severe cases of PPCM are generally seen, there is a possibility of referral bias. Furthermore, considering that PPCM is a rare disease, we acknowledge that the small sample size of this study might have affected the precision of estimates, especially in the logistic regression analyses. We therefore encourage validation of our findings in a larger, multi-centred cohort.

This study also lacks a control group and NT-proBNP measurements at follow-up. The slope of change in NT-proBNP levels in patients with PPCM might provide important information regarding the changes of LV recovery and selection of correct heart failure treatment in future studies.

We acknowledge that RV function, which has previously been shown to have important prognostic value in PPCM, [9] was not assessed in this study. Future studies should evaluate the impact of RV size and function on NT-pro-BNP levels in PPCM.

## Conclusions

We demonstrate the prognostic value of NT-pro-BNP for LV recovery in PPCM. We have shown that NT-proBNP may be useful in the risk stratification of patients with PPCM and may be used to implement more intensive treatment strategies of patients who have an NT-proBNP ≥ 900 pg/ml at diagnosis and individualise follow-up regimens. The application of POC NT-proBNP testing should be further studied for its use of diagnosis and risk stratification for patients with PPCM.
